# Examining Peripheral and Tumor Cellular Immunome in Patients With Cancer

**DOI:** 10.3389/fimmu.2019.01767

**Published:** 2019-07-31

**Authors:** Eda K. Holl, Victoria N. Frazier, Karenia Landa, Georgia M. Beasley, E. Shelley Hwang, Smita K. Nair

**Affiliations:** Department of Surgery, Duke University, Durham, NC, United States

**Keywords:** cancer immunotherapy, cell subsets, cellular immunome, flow cytometry, immune biomarkers, immune monitoring

## Abstract

Immunotherapies are rapidly being integrated into standard of care (SOC) therapy in conjunction with surgery, chemotherapy, and radiotherapy for many cancers and a large number of clinical studies continue to explore immunotherapy alone and as part of combination therapies in patients with cancer. It is evident that clinical effectiveness of immunotherapy is limited to a subset of patients and improving immunotherapy related outcomes remains a major scientific and clinical effort. Understanding the immune cell subset phenotype and activation/functional status (cellular immunome) prior to and post therapy is therefore critical to develop biomarkers that (1) will predict if a patient will respond to immunotherapy and (2) are a result of immunotherapy. In this study, we investigated local (tumor) and peripheral (blood) cellular immunome of patients with melanoma, breast cancer, and brain cancer using a rapid and reliable standardized, multiparameter flow cytometry assay. We used this approach to monitor changes in the peripheral cellular immunome in women with breast cancer undergoing SOC therapy. Our analysis is unique because it is conducted using matched fresh tumor tissue and blood from patients in real-time, within 2–3 h of sample acquisition, and provides insight into the innate and adaptive immune cell profile in blood and tumor. Specific to blood, this approach involves no manipulation and evaluates all immune subsets such as T cells, B cells, natural killer (NK) cells, monocytes, dendritic cells (DCs), neutrophils, eosinophils, and basophils using 0.5 ml of blood. Analysis of the corresponding tumor provides much needed insight into the phenotype and activation status of immune cells, especially T and B cells, in the tumor microenvironment vs. the periphery. This analysis will be used to assess baseline and therapy-mediated changes in local and peripheral cellular immunome in patients with glioblastoma, breast cancer, and melanoma in planned immunotherapy clinical studies.

## Introduction

Over the past 7 years, immunotherapy has dramatically changed the treatment landscape for many solid tumor and hematologic malignancies (1–5). Cancer immunotherapy strategies include cytokines, immune checkpoint blockade, adoptive T cell therapies, vaccines (including dendritic cell (DC) based cellular vaccines) and oncolytic viruses (6–10). In particular, immune checkpoint blockade with antibodies that block CTLA-4, PD-1 or PD-L1 have resulted in durable clinical responses in patients with advanced melanoma, lung, kidney, bladder, and colorectal cancer, but both primary and acquired resistance occur in the majority of patients (1–5, 11). Several components of pre-existing anti-tumor immunity in patients have emerged as key regulators in determining sensitivity to checkpoint blockade (3, 12–14). This suggests that the therapeutic efficacy of immunotherapies can be enhanced with novel combination therapies that promote immunological responses. Novel combination strategies can help overcome the resistance currently seen and may also provide rationale for expansion of these same combination therapies to other solid tumors (15).

Intratumoral injection of an oncolytic virus to promote local recruitment of immune cells to the tumor is being developed as a strategy to overcome resistance to immune blockade (15, 16). We are investigating oncolytic poliovirus (oncPV) therapy, which has demonstrated unprecedented responses in the treatment of recurrent glioblastoma multiforme (GBM), with 21% of patients experiencing durable, long-term (>3 years) survival (17). Although this was remarkably successful given recurrent GBM is a uniformly fatal disease, the majority of patients did not respond to therapy. Therefore, identification of key immune biomarkers is essential to appropriately select patients that can benefit from therapy. These same patterns of cellular immunome may also elucidate the mechanism of tumor responses to the combination of oncPV plus immune checkpoint blockade. The ability to reliably examine the immune cell subset phenotype and activation/functional status (cellular immunome) of cancer patients before and after therapy is critical to the development of predictive markers.

Toward the goal of utilizing the cellular immunome to predict response to immune-based therapies, we analyzed matched blood and tumor tissue from melanoma, breast cancer and brain cancer patients. Previous studies have shown that peripheral immune subtypes can be predictive of intratumoral immune responses and serve as a prognostic factor in cancer patients (18). Analysis of blood and tumor was conducted in real-time, shortly after sample collection with minimal processing to prevent exclusion or death of innate immune cells (e.g., neutrophils, basophils, and eosinophils). This also allows for a true representation of all immune cells in blood and therapy-mediated changes in immune cells. The immune cell subsets that infiltrate the tumors and the percentage of total tumor tissue infiltrated by these cells can also be determined more precisely by immediate processing and analysis of tumor tissue. Additionally, we use pre-cocktailed, dried reagents from the same lot to maintain consistency between samples for every patient throughout therapy. In doing so, we reduce the cost of the process and eliminate operator error. Here we describe a method to query the patient's cellular immunome rapidly during the course of immunotherapy with the goal to advance the development of predictive immune biomarkers that can then be applied as a companion diagnostic in the clinic.

## Materials and Methods

### Patient Recruitment

Eligible patients, 18 years or older, who underwent SOC surgical resection of melanoma, breast cancer, and brain cancer were consented to research tumor tissue and blood collection though the Duke BioRepository and Precision Pathology Center (BRPC) or the Brain Tumor BioRepository (BTBR) at Duke University School of Medicine. All patients were selected based on ability to get both tumor tissue and blood at the same time. Breast cancer patients (*n* = 15) were diagnosed with localized disease and received SOC neoadjuvant chemotherapy followed by surgery. Melanoma patients (*n* = 9) included in this study had stage I-IV disease. Primary tumors or metastatic lymph nodes were resected during SOC therapy. Brain tumor patients (*n* = 3) were newly-diagnosed with grade III glioma or grade IV glioblastoma (GBM) and received SOC surgery. De-identified matched blood and tumor tissue from consented patients was obtained by the BRPC and BTBR and used for examination of the cellular immunome.

### Blood and Tumor Processing

Blood was obtained by venipuncture and collected in Vacutainer collection tubes containing EDTA anticoagulant (BD Biosciences). Blood was rotated on shaker until flow cytometry analysis (10 min-4 h post collection).

Surgically resected tumors were collected and stored in MACS tissue storage solution at 4°C (Miltenyi). Storage time was 2–16 h post-tumor collection. Tumor cells and tumor infiltrating immune cells were analyzed after tumors were processed using the Tumor Dissociation Kit and Gentle MACS mechanical dissociator (Miltenyi) following the manufacturer's recommendations. Single cell suspension was immediately analyzed by flow cytometry.

### Flow Cytometry

All analysis was performed using DuraClone IM (Immune Monitoring) panels: Basic (CD45, CD3, CD4, CD8, CD19, CD14, CD16, CD56), B cell (IgD, CD21, CD19, CD27, CD24, CD38, IgM, CD45), T cell (CD45, CD3, CD4, CD8, CD45RA, CD197/CCR7, CD27, CD28, CD279/PD1, CD57), Granulocyte (CD45, CD294, CD16, CD33, CD11b, CD274, CD3, CD19, CD56, CD14, CD62L, CD15), T cell Receptor (CD45, CD3, CD4, CD8, TCRγδ, TCRαβ, HLA-DR, TCRVδ1, TCRVδ2), Regulatory T cell/Treg (CD45, CD3, CD4, CD45RA, CD25, CD39, Foxp3, Helios), and Dendritic cell/DC (CD45, HLA-DR, CD14, CD19, CD20, CD56, CD16, CD1c, Clec9A, CD123). For peripheral blood analysis, 100 μl of blood was added to the appropriate tubes and cells were processed according to the manufacturer's instructions. In brief, blood was incubated with the antibodies for 15 min in the dark, followed by red blood cell lysis using VersaLyse (Beckman Coulter) for 15 min in the dark. Cells were then washed twice in PBS prior to data acquisition. Treg cell analysis was performed according to the manufacturer's protocol. Briefly, 50 μl of peripheral blood was incubated for 15 min with DuraClone IM Treg Tube 1, followed by a wash in 3 ml of PBS and centrifugation at 500 × g for 5 min. Cells were then resuspended in 50 μl of 100% fetal calf serum followed by 5 μl of fixative reagent. After a 15 min incubation, cells were resuspended in 400 μl of permeabilizing reagent and immediately transferred to DuraClone Treg Tube2 for a 60 min incubation. Cells were then washed in PBS prior to data acquisition. For tumor cells and tumor-infiltrating immune cells, 1 × 10^6^ cells were added to each tube and cell staining was performed as described above for peripheral blood. Tumor cells were filtered three times using a 70 μM cell strainer to remove dead cell debris. All processed samples were then analyzed on a 13-color CytoFlex flow cytometer (Beckman Coulter). Data were analyzed using Kaluza Software (Beckman Coulter).

## Results

**Figure 1:**
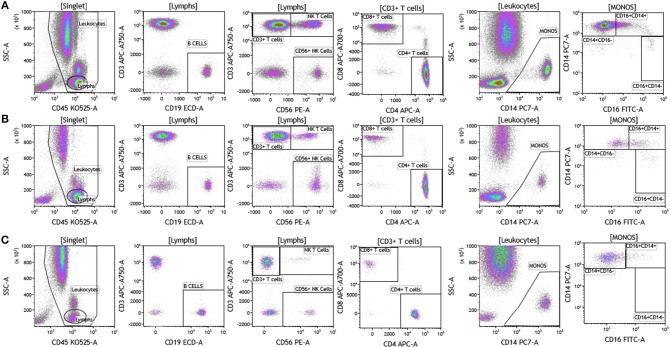
Basic immune cell analysis in peripheral blood of patients with cancer. Whole blood was obtained from cancer patients and analyzed within 2 h of collection (**A**, melanoma; **B**, breast cancer; **C**, brain cancer). Non-single events were excluded based on forward scatter time of flight vs. forward scatter integral. Leukocytes and lymphocytes were gated based on expression of CD45. Cells were separated based on expression of surface markers (CD14, CD16, CD19, CD3, CD56, CD4, and CD8). Different inflammatory stages of monocytes were defined based on CD14 and CD16 cell surface expression, classical monocytes CD14+CD16–, intermediate monocytes CD14+CD16+, and non-classical monocytes CD14–CD16+. Lymphocytes were divided into T cells and B cells based on expression of CD3 (T cells) and CD19 (B cells). NK cells were defined as CD56+ and CD3– and NK T cells were defined as CD56+ and CD3+. CD3+ cells were separated into CD4+ T cells and CD8+ T cells. Range in frequency of cell subsets is shown in Table S1.

We analyzed matched blood and tumor tissue from patients with melanoma, breast cancer, and brain cancer. Figure 1 demonstrates basic panel analysis of whole blood (WB) obtained from melanoma **(1A)**, breast cancer **(1B)**, and brain cancer **(1C)** patients. We examined lymphocytes including B cells (CD19+), CD3+/CD4+ T cells, CD3+/CD8+ T cells, and NK T cells (CD3+CD56+). Total monocytes (CD14+CD16+) (Table S1), were then separated into classical monocytes (CD14+CD16–), intermediate monocytes (CD14+CD16+) and non-classical monocytes (CD14–CD16+). Basic phenotyping analysis revealed that brain tumor patients had 3-8-fold lower percentages of circulating lymphocytes compared to melanoma or breast cancer patients. This was observed in all of the brain cancer patients analyzed, and is in agreement with a recent study demonstrating that patients with newly-diagnosed GBM have very low numbers of T cells in peripheral blood (19).

**Figure 2:**
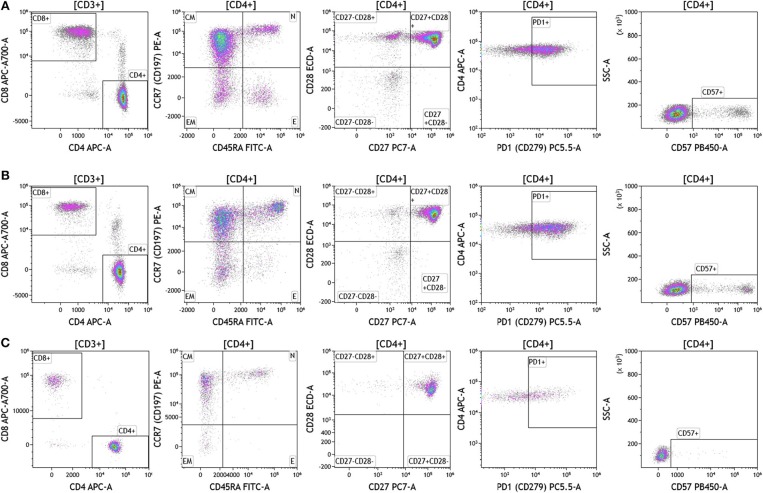
T cell subsets in peripheral blood of patients with cancer. Non-single events were excluded as described in Figure 1 and lymphocytes were gated based on CD45 expression vs. side scatter. **(A)** melanoma; **(B)** breast cancer; **(C)** brain cancer. Gating of T cells was done based on expression of CD3 and side scatter, followed by CD4 and CD8. CD45RA and CCR7 expression was used to identify naïve (CD45RA+CCR7), central memory (CD45RA–CCR7+), effector memory (CD45RA–CCR7–), and effector (CD45RA+CCR7–) T cells. PD1, CD57, CD27, and CD28 expression was examined in CD4 and CD8 T cells. Range in frequency of cell subsets is shown in Table S2. CM, Central Memory; E, effector; EM, Effector Memory; N, naïve.

Duraclone IM T cell panel was used to examine the phenotype and activation status of circulating T cells in melanoma, breast cancer and brain cancer patients (Figure 2 and Table S2). Immune cells were identified using CD45 and side scatter and CD3 was used to separate T cells within the lymphocyte gate followed by analysis of CD4 and CD8 expression within the CD3 subset. The analysis described below was conducted on both CD4 and CD8 T cells. Figure 2 depicts analysis conducted on CD4 T cells and Table S2 provides percentage of individual CD4 and CD8 T cell subsets. Human T cell subsets and differentiation status was assessed using the following cell surface markers: CD45RA, CCR7, CD62L, CD27, and CD28. Loss of CD45RA on naive cells is accompanied by a gain of CD45RO on differentiated cells, thus CD45RA is sufficient to identity naïve vs. differentiated cells (20, 21). In Figure 2, CD4+ T cells were characterized for expression of CD45RA and CCR7 to define naïve (CCR7+CD45RA+), central memory (CCR7+CD45RA–), effector memory (CCR7–CD45RA–), and effector T cells (CCR7–CD45RA+, also referred to as T effector memory cells re-expressing CD45RA or T_EMRA_ cells). CD27 and CD28 (receptors involved in T cell activation) are used to study cellular activation history and are present on naïve and central memory T cells but absent on effector memory and effector T cells (22, 23). Expression of PD1 is normally associated with T cell differentiation and activation, but sustained expression may indicate exhausted T cells in cancer and chronic infections (24). CD57 expression allows the identification of terminally differentiated T cells that have limited proliferative capacity (25). We observed that patients with melanoma had higher percentages of effector T cells (these are CCR7–CD45RA+) as compared to breast and brain cancer patients (Table S2). We therefore examined the effector T cell population in melanoma patients for expression of PD1 and CD57 (markers used to distinguish T effector memory cells re-expressing CD45RA, T_EMRA_, Figure S1) (24). All cells had high expression of both PD1 and CD57 markers which is consistent with T_EMRA_ phenotype suggesting a potential role for this cell type in melanoma.

**Figure 3:**
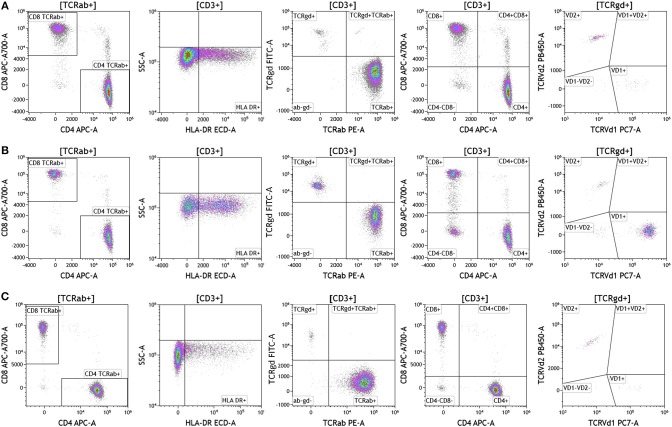
T cell receptor expression in peripheral blood. Blood was analyzed as described in Figures 1, 2. **(A)** melanoma; **(B)** breast cancer; **(C)** brain cancer. HLA-DR expression was used to assess T cell activation. Cells were separated based on surface expression of TCRαβ and TCRγδ. TCRαβ+ cells were then separated into CD4+T cells or CD8+ T cells. TCRγδ were separated into TCRVδ1 and TCRVδ2 expressing cells. Range in frequency of cell subsets is shown in Table S3. TCRab refers to TCRαβ and TCRgd refers to TCRγδ.

Cell surface T cell receptor expression was analyzed in melanoma, breast cancer, and brain cancer patient blood using the DuraClone IM TCR panel (Figure 3 and Table S3). Lymphocytes were identified based on CD3, CD4, and CD8 expression. CD3+ cells were assessed for surface expression of HLA-DR, a marker used to identify activated T cells. CD3+ T cells were then assessed for expression of TCRαβ and TCRγδ. TCRαβ cells account for 95% of all T cells that are found in circulation. TCRγδ cells (which comprise 5% of total T cells) exhibit an innate immune cell-like phenotype and are thought to respond rapidly and broadly to foreign antigens compared to the TCRαβ subtype cells (26). TCRγδ+ T cells were further assessed for expression of TCRVδ1 and TCRVδ2 (27). Interestingly, TCRVδ2 are capable of professional phagocytosis, a characteristic that is not shared by the TCRαβ T cell subset (28). Despite reduced percentages of circulating lymphocytes, brain tumor patients exhibited the highest percentage of TCRVδ2 compared to breast cancer and melanoma patients.

**Figure 4:**
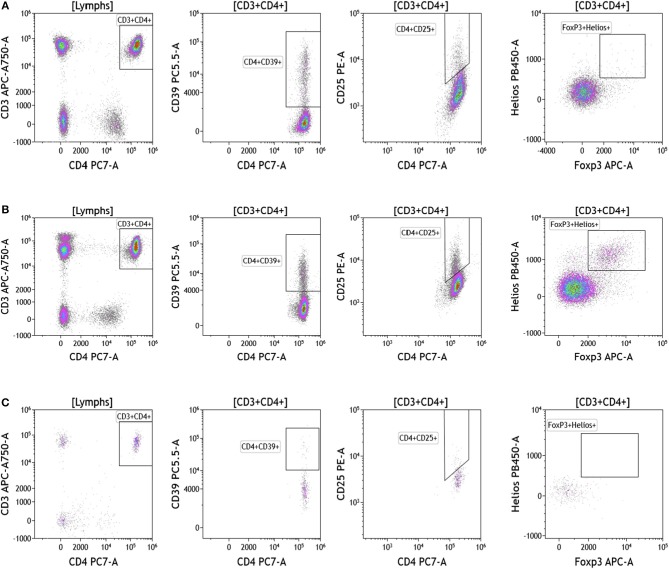
Regulatory T cell analysis in peripheral blood of patients with cancer. Non-single events were excluded as described in Figure 1. **(A)** melanoma; **(B)** breast cancer; **(C)** brain cancer. CD4 T cell populations were identified based on expression of CD45, CD3, and CD4 expression. CD25, Foxp3, Helios1, and CD39 expression was assessed against CD4 expression. Range in frequency of cell subsets is shown in Table S4.

Regulatory T cells (Tregs) and the ratio of regulatory T cells to conventional T cells in blood are used to measure therapy outcomes in patients treated with immunotherapy. To examine Tregs, lymphocytes were identified based on their CD3, CD4, and CD8 expression (Figure 4 and Table S4). CD3+CD4+ T cells were evaluated for Treg markers, including Foxp3, CD25, Helios1, and CD39 (29–33). Treg percentages in blood ranged from 0.83 to 2.83% in melanoma, 3.24 to 9% in breast cancer, and 0.26 to 8.31% in brain tumor.

**Figure 5:**
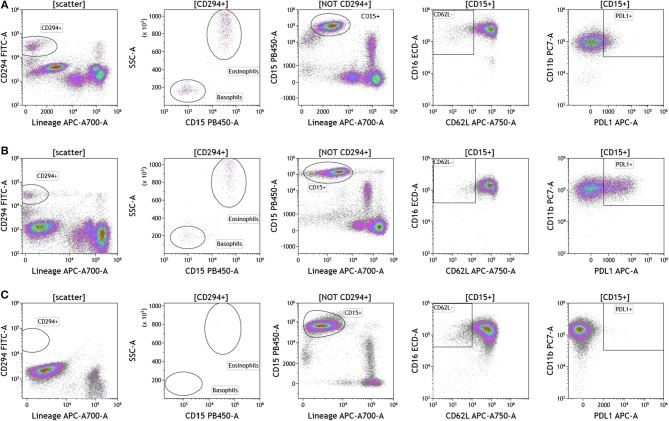
Granulocytes in peripheral blood of patients with cancer. Whole blood from **(A)** melanoma, **(B)** breast cancer, and **(C)** brain cancer patients was analyzed within 2 h of collection. CD294 staining was used to separate basophils and eosinophils from neutrophils. CD62L and PDL1 expression on neutrophils was analyzed to examine neutrophil activation status. Range in frequency of cell subsets is shown in Table S5.

Because we analyzed whole blood, we were able to examine populations that are excluded when analysis is conducted on peripheral blood mononuclear cells (PBMCs), namely granulocytes (Figure 5 and Table S5). Granulocytes, which represent 45–75% of total leukocytes, control inflammatory responses and are the first responders to sites of infection. Granulocytes include neutrophils (45–75%), eosinophils (0–7%), and basophils (0–2%). The resolution of inflammation is directly tied to how long granulocytes, specifically neutrophils, persist in tissues (34). In the context of cancer, neutrophils are known to modulate the tumor microenvironment by exerting both anti-tumor and pro-tumor effects (35). The analysis presented in Figure 5 not only provides an overall assessment of granulocyte composition in blood, but also examines the activation state of neutrophils. Cells that were positive CD294 and negative for lineage markers CD3, CD19, CD56, and CD14 were gated and analyzed for expression of CD15. Eosinophils were characterized as CD294+CD15+ and basophils were characterized CD294+CD15–. Neutrophils were characterized as CD294–CD15+CD3–CD19–CD56–CD14–. Neutrophils were further analyzed for expression of CD62L (adhesion marker that is downregulated upon activation), CD11b (upregulated upon activation), and PDL1 (upregulated upon activation). Our data reveal that breast cancer and melanoma patients had similar percentages of neutrophils, granulocytes, and basophils. The activation status of neutrophils (%PDL1+ cells) was also similar in breast cancer and melanoma patients. Unlike melanoma and breast cancer patients, all brain tumor patients lacked circulating basophils and eosinophils. The absence of eosinophils and basophils was observed in all brain cancer patients. Proportionally, these patients have increased percentages of circulating neutrophils (Table S5).

**Figure 6:**
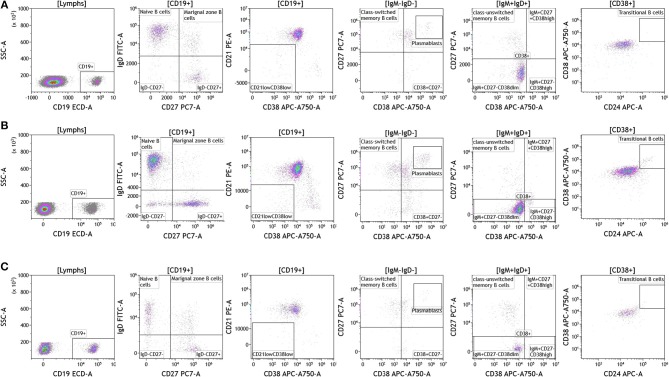
B cell analysis in peripheral blood of patients with cancer. Non-single events were excluded as described in Figure 1. **(A)** melanoma; **(B)** breast cancer; **(C)** brain cancer. B lymphocytes were identified using CD45 and CD19. Naïve and marginal zone B cells were identified based on their expression of IgD and CD27. Class unswitched memory B cells were identified as IgM+IgD+CD27+CD38–. IgM–IgD– B cell were separated into class-switched memory B cells based on expression of CD27 and lack of CD38. Plasmablasts were identified as CD27+CD38+. Range in frequency of cell subsets is shown in Table S6.

Recent studies have highlighted the importance of B cells in cancer, especially in breast cancer (36–41). B cells are known to contribute to antitumor immunity in two ways; produce antitumor antibodies and present antigens to T cells. However, an in-depth analysis of B cells is often not conducted and few studies examine B cell subsets and B cell activation in cancers. We used the DuraClone IM B cell panel to analyze the B cells in the blood of melanoma, breast cancer, and brain cancer patients (Figure 6 and Table S6). B lymphocytes were identified as CD45 and CD19 expressing cells (Figure 6). Their activation status was examined using CD27 and CD28 marker expression. Plasmablasts, which are recently activated antibody producing cells, lack expression of IgM and IgD and express high levels of CD27 and CD38. This analysis identifies naive B cells (IgD+CD27–), marginal zone–like/natural effector B cells (IgD+CD27+), class-switched memory B cells (IgD–CD27+), CD21^low^CD38^low^ B cells, transitional B cells (IgM+CD38+), and plasmablasts (IgM–CD38+). Increased numbers of memory B cells and plasmablasts in blood and tumor would indicate B cell responses in patients with cancer (42). This would also highlight the need to examine changes in B cell receptor (BCR) clonality and diversity in patients with cancer undergoing therapy.

**Figure 7:**
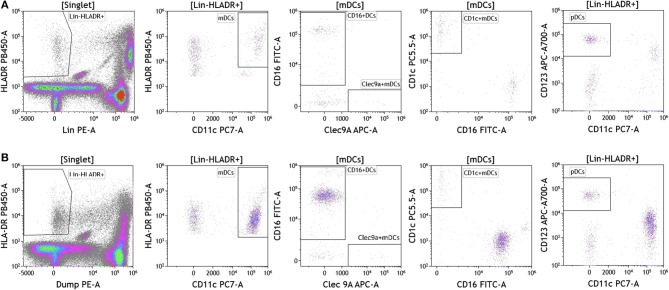
Dendritic cell analysis in peripheral blood of patients with cancer. Non-single events were excluded as described in Figure 1. **(A)** melanoma; **(B)** breast cancer; **(C)** brain cancer. DCs were identified based on expression of HLA-DR and lack of lineage (Lin) markers (CD3, CD14, CD19, CD20, CD56). Myeloid DCs (mDCs) were identified as Lin-HLADR+CD11c+. All mDCs were further divided CD11c+CD1c+CD16– mDC1, CD11c+Clec9A+ CD16– mDC2 and CD11c+CD16+Clec9A– inflammatory mDCs. pDCs were identified as HLADR+CD11c–CD123+. Range in frequency of cell subsets is shown in Table S7. DC, dendritic cell; mDC, myeloid dendritic cell; pDC, plasmacytoid dendritic cell.

Dendritic cells (DCs) are potent antigen presenting cells (43) and have been used in multiple clinical trials across tumor types as cellular vaccines. Although DCs represent 1% or less of circulating lymphocytes, the importance of DC analysis in blood cannot be understated given their innate ability to respond to pathogen- and danger-associated molecular patterns and consequently their ability to stimulate naïve T and B lymphocytes. It is important to examine DC subsets and changes in DC subset frequency because DCs are critical in immunostimulation and immunoregulation. Using the DuraClone IM DC panel, we examined DC populations in the peripheral blood of cancer patients (Figure 7 and Table S7). We gated on HLA-DR+ (class II+) cells that were negative for all lineage (Lin) markers (CD3, CD14, CD19, CD20, CD56). These cells were then analyzed based on expression of the CD11c surface marker to identify HLA-DR+CD11c+ myeloid DCs (mDCs) and HLA–DR+CD11c– plasmacytoid DCs (pDCs). All mDCs were further divided into DC subsets as follows: CD11c+Clec9A+CD16– (normally 0.02–0.06% of PBMCs), CD11c+CD1c+CD16– (normally 0.3–0.8% of PBMCs), and CD11c+CD16+Clec9A– inflammatory mDCs (normally 0.75–2% of PBMCs). Expression of CD123 was used to confirm pDC phenotype (normally 0.3–0.8% of PBMCs). Although circulating DCs were found in both melanoma and breast cancer patients, brain tumor patients lacked all DCs in circulation and flow plots are therefore not presented in Figure 7. Interestingly, melanoma patients had an increased average of pDCs in circulation. pDCs are known to play an important role in melanoma and are actively being studied for their potential to induce type I IFN production and antitumor immunity (44).

**Figure 8:**
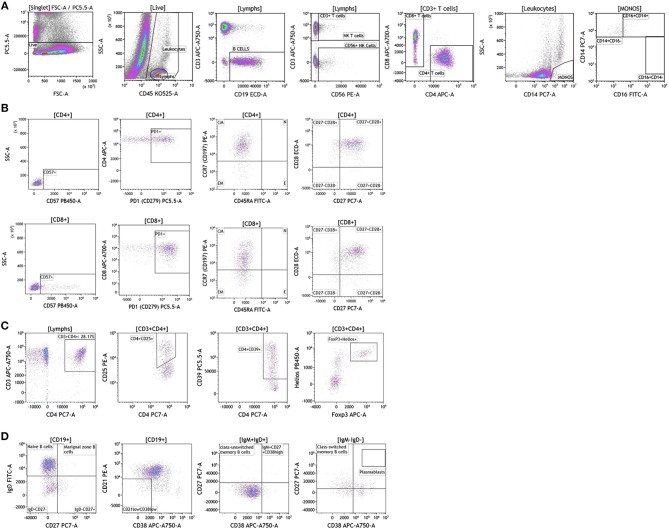
Immune cell analysis in melanoma tumor tissue. Melanoma tumors were gated on live cells (PI-negative). Immune cell analysis was then carried out as described in Figure 1. **(A)** Basic immune cell analysis; **(B)** T cell subset analysis; **(C)** Treg analysis. **(D)** B cell analysis. Range in frequency of cell subsets is shown in Tables S8, S9. PI; propidium iodide. CM, Central Memory; E, effector; EM, Effector Memory; N, naïve.

Next we examined the local (tumor) cellular immunome in patients with melanoma, breast cancer and brain cancer. Surgically resected fresh tumor tissue (matched to blood from same patient) was processed into a single cell suspension within 16 h of tumor harvest followed immediately by flow cytometry. Figure 8 shows analysis of melanoma tumors, basic analysis of all tumor-infiltrating lymphocytes, T cell subset, Treg, and B cell analysis. Dead cells were excluded and CD45 was used to distinguish immune cells from tumor cells followed by examination of CD4+ and CD8+ T cells, CD19+ B cells and CD56+ NK T cells. We collected data from a total of nine patients presenting with localized stage I disease, stage IIB, stage IIIC, and metastatic stage IV disease. In Figure 8A we show overall composition of immune cell infiltrates found in a representative melanoma tumor. Overall lymphocyte infiltration in the analyzed tumors varied between 2.02 and 39.12% of total live cells. CD3+ T cells accounted for the majority of infiltrating lymphocytes 54.77–89.95%. In the shown example, B cells accounted for 36% of all lymphocytes. This large B cell infiltration was only observed in two of the patients. Three out of nine tumors showed low percentages of infiltrating B cells (~3–6% of infiltrating lymphocytes) and the range in the other four tumors tested was 9–17% of all lymphocytes. Overall monocyte percentages were low in all of the tumors analyzed and the largest percentage of these cells were of the classical or intermediate phenotype (Table S8). NK Cells and NK T cells observed in these tumors are also presented in Table S8. These percentages varied greatly between the tumors analyzed.

We analyzed T cell subsets to examine the phenotype and differentiation status of these T cells. In Figure 8B and Table S9, we show analysis of both CD4+ and CD8+ T cell subsets. Both subtypes had similar overall expression of CD57. PD1 expression was elevated in both T cell subsets, which is in agreement with previously published studies in melanoma patients (45). This discovery is not surprising given the success that immunotherapy treatments have had in melanoma patients with pre-existing anti-tumor immunity. Further analysis revealed that both T cell subsets express CCR7 and lack CD45RA, a hallmark of central memory T cells. These cells were also positive for both CD27 and CD28 cell-surface markers. Taken together these data show that although T cells are abundant in melanoma tumors, these cells display a central memory phenotype with significant expression of PD1.

Regulatory T cells are an important CD4+ T cell subtype that can impact anti-tumor immune responses (46). In the shown analysis, regulatory T cells accounted for ~50% of all CD4+ T cells (Figure 8C), a phenotype that potentially contributes to anti-cancer therapy resistance.

In the two melanoma patients with high percentage of B cell infiltrates in the tumor, we further phenotyped the infiltrating B cell subpopulations. Data presented in Figure 8D demonstrates that these cells primarily display a naïve B cell phenotype. Plasmablasts and transitional B cells were scarce. It is unclear if these cells display a regulatory B cell phenotype. These studies are ongoing as part of a larger initiative and will be reported in a separate manuscript.

**Figure 9:**
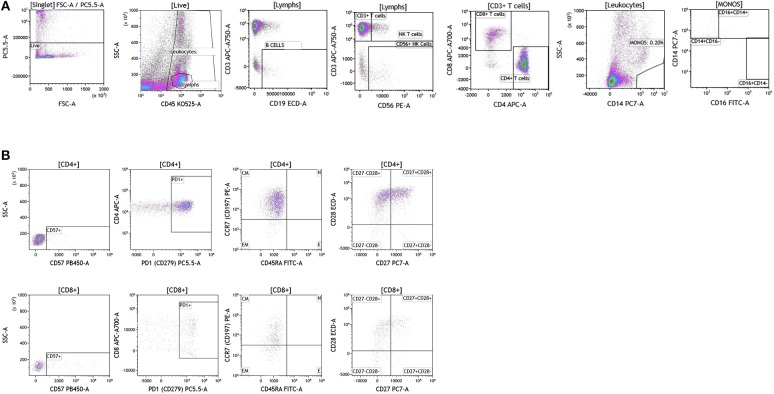
Immune cell analysis in breast tumor tissue. Breast tumors were gated on live cells (PI-negative). Immune cell analysis was then carried out as described in Figure 1. **(A)** Basic immune cell analysis; **(B)** T cell subset analysis. Range in frequency of cell subsets is shown in Tables S8, S9. CM, Central Memory; E, effector; EM, Effector Memory; N, naïve. PI; propidium iodide.

Breast cancer analysis was conducted with tumor tissue from 15 women presenting with localized disease who underwent surgery as part of SOC therapy. We processed all samples fresh in order to avoid cell death and cell surface marker alteration as a result of the freeze-thawing cycle (47). In Figure 9 we show overall flow cytometry analysis of infiltrating immune cells in a representative example. T cells account for the majority of infiltrating lymphocytes in all tumors (average and percentage ranges are shown in Table S8). Of all T cells, CD4 T cells were the predominant subpopulation observed in 11 out of 15 tumors. B cells (>10% of all lymphocytes) were present in five out of the 15 tumors tested. The remaining infiltrating immune cells were monocytes, NK cells, and NK T cells. It is important to note that immune cell infiltration in the majority of the breast tumors examined was relatively low (1.59–10% of all live cells found in the tumor).

Next we analyzed CD4+ and CD8+ T cells for expression of CD57 and PD1. Data presented in Figure 9B and Table S9 show CD57 expression percentages in the analyzed breast tumors. PD1 expression was high in all of the tumor infiltrating T cells that were analyzed (>60.22 of all CD4+ or CD8+ T cells) suggesting exhaustion of these infiltrating cells and a potential role for checkpoint therapy in breast tumors with pre-existing infiltrating T cells. These cells displayed a central memory phenotype, similar to T cells observed in melanoma patients. Unlike CD4+ and CD8+ T cells observed in melanoma patients, T cells observed in breast tumors showed differential expression of the CD27 cell surface marker, where only half of the CD28+ cells were also positive for cell-surface CD27. Although a role has not been assigned to this cell population in breast cancer, previous studies in other tumor types suggest that CD27 co-expression on the surface of CD8+ T cells directly correlates with the ability of these CD8+ T cells to mediate tumor regression (48).

We were able to conduct a comprehensive analysis of the T cell subset in breast tumors obtained from patients who underwent SOC therapy, however, our analysis was limited with regards to B cell analysis. Although, a fraction of the breast tumors were infiltrated by significant numbers of B cells, we lacked sufficient tumor sample to complete a detailed B cell analysis. In future studies, we plan to focus specifically on the B cell subsets that are found in breast tumors.

**Figure 10:**
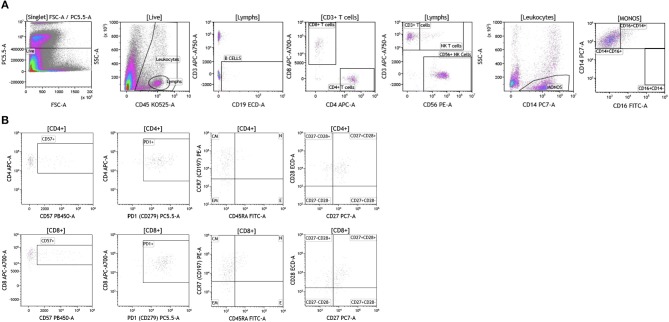
Immune cell analysis in brain tumor tissue. Brain tumors were gated on live cells (PI-negative). Immune cell analysis was then carried out as described in Figure 1. **(A)** Basic immune cell analysis; **(B)** T cell subset analysis. Range in frequency of cell subsets is shown in Tables S8, S9. PI; propidium iodide. CM, Central Memory; E, effector; EM, Effector Memory; N, naïve.

Brain tumor immune analysis was conducted in three patients whose tumors were resected as part of SOC therapy. In Figure 10 we show a representative example of the immune infiltrates in brain tumors. Brain tumors were characterized by low percentages of infiltrating immune cells (Table S8). Lymphocytes accounted for <2% of all analyzed cells. This finding is not surprising given the low numbers of circulating lymphocytes in these patients and recent reports showing lymphocyte sequestration of T cells in the bone marrow of brain tumor patients (19). Unlike other tumor types analyzed, brain tumors showed high percentages of infiltrating NK cells (14.23–50.45% of all infiltrating lymphocytes), which suggests a potential role for this cell type in brain cancer.

Analysis of T cell subsets in brain tumor revealed that infiltrating CD4+ and CD8+ T cells expressed CD57 (Table S9). PD1 was also highly expressed on both CD4+ and CD8+ T cell subtypes suggesting T cell exhaustion in these tumors. Unlike the other tumor types analyzed, T cells infiltrating brain tumors display an effector memory phenotype based on their lack of CCR7 and CD45RA expression. Further analysis in larger cohort of brain tumor patients is currently ongoing in the context of immunotherapy clinical trials to examine how and which immune infiltrating cells impact therapeutic outcomes.

We analyzed melanoma, breast, and brain tumors for the presence of granulocytes and dendritic cells and observed that these cells were not present in the tumors.

**Figure 11:**
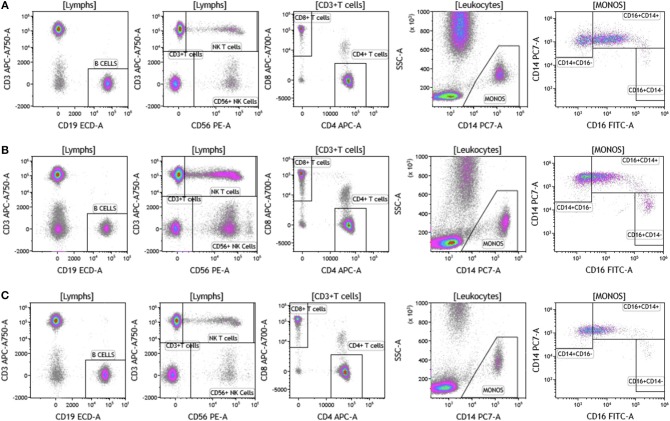
Basic immune cell analysis in blood of women with breast cancer undergoing standard of care therapy. Blood was obtained from women undergoing standard of care therapy (chemotherapy and surgery) for breast cancer; **(A)** prior to therapy; **(B)** post-chemotherapy and pre-surgery; **(C)** 2-months post-surgery follow-up. The same patient was followed at each time point as described in Figure 1. Range in frequency of cell subsets is described in Table S10. Data are representative of analysis conducted in three women with breast cancer.

The analysis that we have presented was developed to examine baseline and therapy-mediated changes in the peripheral (blood) cellular immunome in the context of therapy along with companion analysis in tumors before and after therapy when feasible. Therefore, to validate our peripheral cellular immunome analysis, we longitudinally examined blood from women with breast cancer undergoing SOC therapy. Analysis of blood was performed as described above using the DuraClone IM basic panel (Figure 11). Blood was obtained prior to chemotherapy (Figure 11A), post-chemotherapy at the time of surgery (Figure 11B) and 2 months after surgery (Figure 11C). As shown in Table S10, we observed changes in the peripheral cellular immunome during the course of treatment. The percentage of natural killer cells doubled post chemotherapy (at the time of surgery) but returned to pre-therapy levels 2 months after surgery. Non-classical monocytes (CD16+CD14-) increased >4-fold and the percentage of natural killer cells doubled post-chemotherapy (at the time of surgery) but both returned to pre-therapy levels 2 months after surgery. This preliminary analysis suggests that we can capture changes in the peripheral cellular immunome. Such an analysis will accurately allow us to monitor the baseline cellular immunome and longitudinal changes in all cellular subsets (including granulocytes) during the course of SOC chemotherapy, surgery, and immunotherapy. The reproducibility of this analysis is guaranteed because we use pre-standardized dried reagents from the same lot to allow precise measurement of changes in the cellular immunome.

## Discussion

The ability to precisely and reproducibly understand immune cell phenotype and activation status (cellular immunome) in tumor and blood is critical to assess if a patient has responded to therapy and to develop biomarkers of response to therapy. The analysis presented in this manuscript was done using paired tumor and blood samples from patients with cancer with the following objectives: (1) examine blood and tumor cellular immunome in patients with cancer; (2) determine feasibility of this approach across different tumor types, and (3) assess if the experimental protocol is reproducible and precise to allow longitudinal monitoring of the peripheral cellular immunome to measure changes in response to therapies. Although cost of real time analysis can be a concern during immune monitoring, a side by side comparison between real times analysis of samples and analysis of frozen PBMCs revealed comparable overall costs.

In this study we present analysis conducted in patients with melanoma, breast cancer, and brain cancer. Our data demonstrate that we can indeed reliably and accurately monitor patient cellular immunome in tumor and blood in a matter of 2–5 h (blood) and 2–16 h (tumor) after sample acquisition and get a complete profile that encompasses 77 immune subsets. The value of developing an assay that has a quick turn-around and can be conducted in real-time is important in biomarker development; specifically if we want to use blood and tumor cellular immunome to drive decisions related to therapy. Conducting analysis of blood in real-time is important for many reasons. The use of fresh blood eliminates the need to purify and freeze peripheral blood mononuclear cells (PBMCs), which introduces variability during isolation, freezing, storage, and thawing procedures. The use of whole blood permits analysis of all cell populations in blood (including neutrophils, eosinophils, and basophils). For example, our analysis of granulocytes in Figure 5 provides a broad view of the complete profile of the peripheral cellular immunome profile, given that neutrophils represent ~45–75% of all leukocytes. The analysis provides information on neutrophil activation status and PDL1 expression. PDL1 expression on neutrophils in patients ranged from 5.04 to 31.24% in breast cancer and 11.77–49.65% in melanoma (Table S5) suggesting that these immunoregulatory PDL1+ neutrophils may have an immunosuppressive role. Luo et al. demonstrated that PDL1+ neutrophils were elevated in patients with rheumatoid arthritis (49). A recent study demonstrated a negative correlation between neutrophil and CD8 T cell levels in patients with non-small cell lung cancer (50).

Our data in Figures 1–11 and Tables S1–S10 provides a snapshot of the local (tumor) and peripheral (blood) cellular immunome in patients with melanoma, breast tumor, brain tumor. We also saw qualitative differences in our samples, for example lack of eosinophils and basophils and fewer lymphocytes in patients with brain tumor. These qualitative differences are highlighted in the results section in the context of the cell subset examined. These changes could be in response to ongoing therapies and studies to analyze these changes are currently underway. For example, we are currently investigating therapy-induced changes in women with breast cancer (*n* = 100). Specifically, we are monitoring the peripheral cellular immunome at baseline, post-chemotherapy, and pre-surgery and 2 months post-surgery to comprehensively address how the peripheral cellular immunome is impacted during SOC therapy. Also part of this planned study is the investigation of the local/tumor cellular immunome, in patients undergoing SOC surgery. An example of longitudinal analysis of the peripheral cellular immunome in women with breast cancer undergoing SOC therapy is presented in Figure 11. Here we present the basic phenotype panel analysis at three time-points: baseline, post-chemotherapy, and pre-surgery and 2 months post-surgery. Our analysis revealed an increase in NK cells and NKT cells, post-chemotherapy and these numbers returned to pre-therapy levels 2 months post-surgery. The monocyte compartment also changed with therapy. The monocyte compartment in whole blood has three subsets: CD14+CD16- classical monocytes (phagocytosis), CD14+CD16+ intermediate monocytes (pro-inflammatory, phagocytosis) and CD14-CD16+ non-classical monocytes (patrolling, antiviral, pro-inflammatory) (51, 52). As shown in Figure 11 and Table S10, we observed an increase in non-classical monocytes post-treatment (presumably indicating inflammation following chemotherapy or surgery) and return to pre-therapy levels 2 months after surgery. An increase in the number of CD4+CD8+ T cells was also observed post-treatment (Figures 11A–C; panel 3, top right), with a return to pre-therapy numbers post-therapy. As such, double positive CD4 and CD8 T cells have been described in both healthy donors, patients with inflammatory disorders and patients with cancer, including breast cancer (53). What is not completely clear is the function of these T cells as they have been reported to be suppressive or cytotoxic. Clearly our next task is to elucidate the function of these cells and such studies are planned.

In ongoing studies we are also developing immune function analysis using whole blood. This ensures immune function analysis is done in the context of all cell types in blood, for example platelets, or soluble serum factors that may be relevant to immune function. Studies suggest that whole blood function more accurately represents *in vivo* immune competence than PBMC function (54–56). We did not examine the function of monocytes, NK cells, and T cells in this study. We have developed these panels and will present our findings in a follow-up study. Lastly, this study does not describe the macrophage compartment in the tumors. As such, the presence and relevance of macrophages in human tumors has been a subject of considerable research (57–59). We did observe the presence of CD14+ and CD16+ cells that displayed high side scatter within the tumors (Figure S2), which may be either macrophages or Tie-2-expressing monocytes (60, 61). To elucidate this further, a macrophage-specific panel is being developed for melanoma, breast, and brain tumors (this panel includes antibodies to CD45, CD11b, CD16, CD14, CD68, CD163, HLA-DR, and CD66b). We plan to conduct a focused analysis of macrophages in 10–15 tumor tissues for each of these cancers and perform parallel immunohistochemistry (IHC) analysis to examine macrophages in the context of tumor microenvironment.

These studies were initiated to develop a robust method for cellular immunome monitoring in the context of our current immunotherapy clinical trials. In a recent clinical study with a novel recombinant oncolytic poliovirus, PVSRIPO, we reported unprecedented responses in the treatment of recurrent glioblastoma, with 21% of patients experiencing durable, long-term (>3 years) survival (17). Our team also demonstrated that the oncolytic poliovirus, PVSRIPO, infects, and kills tumor cells while simultaneously activating innate immune cells, which leads to efficient priming of adaptive antitumor immune response (62). In the clinical study in patients with recurrent glioblastoma (17), 79% of patients did not respond to PVSRIPO, making the identification of biomarkers that predict response to therapy and mechanisms of resistance critical areas of research. The development of rapid, reliable, and reproducible monitoring of the cellular immunome is required for immune biomarker development. We will use the analysis presented in this study in the planned clinical studies in patients with recurrent glioblastoma (ClinicalTrials.gov Identifier: NCT02986178), breast cancer (ClinicalTrials.gov Identifier: NCT03564782), and melanoma (ClinicalTrials.gov Identifier: NCT03712358).

## Ethics Statement

This study was approved by the Duke Institutional Review Board as an exempt protocol to access blood and human tissue through the Duke BioRepository and Precision Pathology Center (BRPC) or the Brain Tumor BioRepository (BTBR).

## Author Contributions

EKH designed study, administered the project, performed and analyzed experiments, and wrote manuscript. VF and KL performed and analyzed experiments and reviewed manuscript. GB provided clinical samples and edited manuscript. ESH provided clinical samples and reviewed manuscript. SN designed study, administered the project, analyzed experiments, and wrote manuscript.

### Conflict of Interest Statement

The authors declare that the research was conducted in the absence of any commercial or financial relationships that could be construed as a potential conflict of interest.

## Supplementary Material

Figure S1 PD1 and CD57 expression on effector T cells in melanoma patients. CD4+ T cells that expressed CD45RA but lacked CCR7 were further examined for PD1 and CD57 cell surface expression.

Figure S2 CD14+SSChi cells express CD14 and CD16. **(A)** melanoma; **(B)** breast cancer; **(C)** brain cancer. Tumors immune infiltrates from melanoma, breast, and brain tumor patients were analyzed as shown in Figures 8–10. All cells that were positive for CD14 with high SSC (side scatter) and distinct from blood monocytes were assessed for expression of CD14 and CD16.

Table S1 The frequency of leukocytes (as percentage of singlets), lymphocytes (as percentage of singlets), CD19+ B cells, CD3+ T cells, NK T cells and NK Cells (as percentage of all lymphocytes), CD4+ and CD8+ T cells (as percentage of CD3+ T cells), monocytes (as percentage of all leukocytes) and monocyte subsets (as percentage of all monocytes). Data shows average for all patients and low-high range.

Table S2 The frequency of CD4+ or CD8+ T cell subsets (as percentage of CD4+ or CD8+ T cells). Data shows average for all patients and low-high range.

Table S3 The frequency of HLA-DR+ T cells (as percentage of CD3+ T cells), TCRαβ and TCRγδ T cells (as percentage of CD3+ T cells), CD4+TCRαβ+ and CD8+TCRαβ+ T cells (as percentage of TCRαβ+ T cells), Vδ1-Vδ2-, Vδ1+, Vδ2+, and Vδ1+Vδ2+ (as percentage of all TCRγδ+ T cells). Data shows average for all patients and low-high range.

Table S4 The frequency of CD3+CD4+ T cells (as percentage of all lymphocytes), CD39+, CD25+ or FoxP3+Helios+ subsets (as percentage of CD4+ T cells). Data shows average for all patients and low-high range.

Table S5 The frequency of CD294+ cells (as percentage of singlets), basophils and eosinophils (as percentage of all CD294+ cells), CD15+ cells (as percentage of all cells without CD294+ cells), CD62L– (as percentage of CD15+ cells), PDL1+ (as percentage of CD15+ cells). Data shows average for all patients and low-high range.

Table S6 The frequency of B cell subsets (as percentage of CD19+ B cells), class-switched memory B cells, CD27– CD38–, CD27+CD38+, CD38+CD27- (as percentage of all IgM–IgD– CD19+ B cells), class-unswitched memory B cells, IgM+CD27+CD38^high^, IgM+CD27-CD38^dim^, IgM+CD27–CD38^dim^ (as percentage of all IgM+IgD+ CD19+ B cells), transitional B cells (as percentage of all CD38+ CD19+ B cells). Data shows average for all patients and low-high range.

Table S7 The frequency of Lin-HLADR+ cells (as percentage of singlets), pDCs (as percentage of Lin-HLADR+ cells), mDCs (as percentage of Lin-HLADR+ cells), CD16+, CD11c+Clec9A+ CD16-, and CD11c+CD1c+CD16-subsets (as percentage of mDCs). Data shows average for all patients and low-high range.

Table S8 The frequency of leukocytes (as percentage of singlets), lymphocytes (as percentage of singlets), CD19+ B cells, CD3+ T cells, NK T cells and NK Cells (as percentage of all lymphocytes), CD4+ and CD8+ T cells (as percentage of CD3+ T cells), monocytes (as percentage of all leukocytes), and monocyte subsets (as percentage of all monocytes). Data shows average for all patients and low-high range.

Table S9 The frequency of CD4+ or CD8+ T cell subsets (as percentage of CD4+ or CD8+ T cells). Data shows average for all patients and low-high range.

Table S10 The frequency of leukocytes (as percentage of singlets), lymphocytes (as percentage of singlets), CD19+ B cells, CD3+ T cells, NK T cells and NK Cells (as percentage of all lymphocytes), CD4+ and CD8+ T cells (as percentage of CD3+ T cells), monocytes (as percentage of all leukocytes) and monocyte subsets (as percentage of all monocytes). Data shows average for all patients and low-high range. The patients presented in this study had varying degrees of response to chemotherapy. Patient 1 exhibited a complete pathologic response, patient 2 had a partial response to chemotherapy with sub-millimeter foci of residual tumor in the tumor bed and patient 3 had a minimal response to chemotherapy.

## References

[1] Topalian SL Sznol M McDermott DF Kluger HM Carvajal RD Sharfman WH. Survival, durable tumor remission, and long-term safety in patients with advanced melanoma receiving nivolumab. J Clin Oncol. (2014) 32:1020–30. 10.1200/JCO.2013.53.0105 24590637 PMC4811023

[2] Brahmer J Reckamp KL Baas P Crino L Eberhardt WE Poddubskaya E. Nivolumab versus docetaxel in advanced squamous-cell non-small-cell lung cancer. N Engl J Med. (2015) 373:123–35. 10.1056/NEJMoa1504627 26028407 PMC4681400

[3] Taube JM Klein A Brahmer JR Xu H Pan X Kim JH. Association of PD-1, PD-1 ligands, and other features of the tumor immune microenvironment with response to anti-PD-1 therapy. Clin Cancer Res. (2014) 20:5064–74. 10.1158/1078-0432.CCR-13-3271 24714771 PMC4185001

[4] O'Donnell JS Long GV Scolyer RA Teng MW Smyth MJ. Resistance to PD1/PDL1 checkpoint inhibition. Cancer Treat Rev. (2017) 52:71–81. 10.1016/j.ctrv.2016.11.007 27951441

[5] Sharma P Hu-Lieskovan S Wargo JA Ribas A. Primary, adaptive, and acquired resistance to cancer immunotherapy. Cell. (2017) 168:707–23. 10.1016/j.cell.2017.01.017 28187290 PMC5391692

[6] Ribas A Wolchok JD. Cancer immunotherapy using checkpoint blockade. Science. (2018) 359:1350–5. 10.1126/science.aar4060 29567705 PMC7391259

[7] Chapuis AG Lee SM Thompson JA Roberts IM Margolin KA Bhatia S. Combined IL-21-primed polyclonal CTL plus CTLA4 blockade controls refractory metastatic melanoma in a patient. J Exp Med. (2016) 213:1133–9. 10.1084/jem.20152021 27242164 PMC4925025

[8] Carreno BM Magrini V Becker-Hapak M Kaabinejadian S Hundal J Petti AA. Cancer immunotherapy. A dendritic cell vaccine increases the breadth and diversity of melanoma neoantigen-specific T cells. Science. (2015) 348:803–8. 10.1126/science.aaa3828 25837513 PMC4549796

[9] Andtbacka RH Kaufman HL Collichio F Amatruda T Senzer N Chesney J. Talimogene laherparepvec improves durable response rate in patients with advanced melanoma. J Clin Oncol. (2015) 33:2780–8. 10.1200/JCO.2014.58.3377 26014293

[10] Knutson KL Disis ML. Tumor antigen-specific T helper cells in cancer immunity and immunotherapy. Cancer Immunol Immunother. (2005) 54:721–8. 10.1007/s00262-004-0653-2 16010587 PMC11032889

[11] Postow MA Callahan MK Wolchok JD. Immune checkpoint blockade in cancer therapy. J Clin Oncol. (2015) 33:1974–82. 10.1200/JCO.2014.59.4358 25605845 PMC4980573

[12] Overacre-Delgoffe AE Chikina M Dadey RE Yano H Brunazzi EA Shayan G. Interferon-gamma drives treg fragility to promote anti-tumor immunity. Cell. (2017) 169:1130–41.e11. 10.1016/j.cell.2017.05.005 28552348 PMC5509332

[13] Wistuba-Hamprecht K Martens A Heubach F Romano E Geukes Foppen M Yuan J. Peripheral CD8 effector-memory type 1 T-cells correlate with outcome in ipilimumab-treated stage IV melanoma patients. Eur J Cancer. (2017) 73:61–70. 10.1016/j.ejca.2016.12.011 28167454 PMC5599126

[14] Auslander N Zhang G Lee JS Frederick DT Miao B Moll T Robust prediction of response to immune checkpoint blockade therapy in metastatic melanoma. Nat Med. (2018) 24:1942 10.1038/s41591-018-0157-9 PMC9639231 30333558

[15] Ribas A Dummer R Puzanov I VanderWalde A Andtbacka RHI Michielin O. Oncolytic virotherapy promotes intratumoral T cell infiltration and improves anti-PD-1 immunotherapy. Cell. (2017) 170:1109–19.e10. 10.1016/j.cell.2017.08.027 28886381 PMC8034392

[16] Zamarin D Holmgaard RB Subudhi SK Park JS Mansour M Palese P. Localized oncolytic virotherapy overcomes systemic tumor resistance to immune checkpoint blockade immunotherapy. Sci Transl Med. (2014) 6:226ra32. 10.1126/scitranslmed.3008095 24598590 PMC4106918

[17] Desjardins A Gromeier M Herndon JE II Beaubier N Bolognesi DP Friedman AH. Recurrent glioblastoma treated with recombinant poliovirus. N Engl J Med. (2018) 379:150–61. 10.1056/NEJMoa1716435 29943666 PMC6065102

[18] Niccolai E Cappello P Taddei A Ricci F D'Elios MM Benagiano M. Peripheral ENO1-specific T cells mirror the intratumoral immune response and their presence is a potential prognostic factor for pancreatic adenocarcinoma. Int J Oncol. (2016) 49:393–401. 10.3892/ijo.2016.3524 27210467

[19] Chongsathidkiet P Jackson C Koyama S Loebel F Cui X Farber SH. Sequestration of T cells in bone marrow in the setting of glioblastoma and other intracranial tumors. Nat Med. (2018) 24:1459–68. 10.1038/s41591-018-0135-2 30104766 PMC6129206

[20] Appay V van Lier RA Sallusto F Roederer M. Phenotype and function of human T lymphocyte subsets: consensus and issues. Cytometry A. (2008) 73:975–83. 10.1002/cyto.a.20643 18785267

[21] Mahnke YD Brodie TM Sallusto F Roederer M Lugli E. The who's who of T-cell differentiation: human memory T-cell subsets. Eur J Immunol. (2013) 43:2797–809. 10.1002/eji.201343751 24258910

[22] Larbi A Fulop T From “truly naive” to “exhausted senescent” T cells: when markers predict functionality. Cytometry A. (2014) 85:25–35. 10.1002/cyto.a.22351 24124072

[23] Caorsi C Niccolai E Capello M Vallone R Chattaragada MS Alushi B. Protein disulfide isomerase A3-specific Th1 effector cells infiltrate colon cancer tissue of patients with circulating anti-protein disulfide isomerase A3 autoantibodies. Transl Res. (2016) 171:17-28 e1-2. 10.1016/j.trsl.2015.12.013 26772958

[24] Fuertes Marraco SA Neubert NJ Verdeil G Speiser DE. Inhibitory Receptors Beyond T Cell Exhaustion. Front Immunol. (2015) 6:310. 10.3389/fimmu.2015.00310 26167163 PMC4481276

[25] Kared H Martelli S Ng TP Pender SL Larbi A. CD57 in human natural killer cells and T-lymphocytes. Cancer Immunol Immunother. (2016) 65:441–52. 10.1007/s00262-016-1803-z 26850637 PMC11029668

[26] Holtmeier W Kabelitz D. gammadelta T cells link innate and adaptive immune responses. Chem Immunol Allergy. (2005) 86:151–83. 10.1159/000086659 15976493

[27] Adams EJ Gu S Luoma AM. Human gamma delta T cells: evolution and ligand recognition. Cell Immunol. (2015) 296:31–40. 10.1016/j.cellimm.2015.04.008 25991474 PMC4466157

[28] Wu Y Wu W Wong WM Ward E Thrasher AJ Goldblatt D. Human gamma delta T cells: a lymphoid lineage cell capable of professional phagocytosis. J Immunol. (2009) 183:5622–9. 10.4049/jimmunol.0901772 19843947

[29] Deaglio S Dwyer KM Gao W Friedman D Usheva A Erat A. Adenosine generation catalyzed by CD39 and CD73 expressed on regulatory T cells mediates immune suppression. J Exp Med. (2007) 204:1257–65. 10.1084/jem.20062512 17502665 PMC2118603

[30] Lin X Chen M Liu Y Guo Z He X Brand D. Advances in distinguishing natural from induced Foxp3(+) regulatory T cells. Int J Clin Exp Pathol. (2013) 6:116–23. 23329997 PMC3544233

[31] Rodriguez-Perea AL Arcia ED Rueda CM Velilla PA. Phenotypical characterization of regulatory T cells in humans and rodents. Clin Exp Immunol. (2016) 185:281–91. 10.1111/cei.12804 27124481 PMC4991523

[32] Valmori D Merlo A Souleimanian NE Hesdorffer CS Ayyoub M. A peripheral circulating compartment of natural naive CD4 Tregs. J Clin Invest. (2005) 115:1953–62. 10.1172/JCI23963 16007258 PMC1159133

[33] Niccolai E Ricci F Russo E Nannini G Emmi G Taddei A The different functional distribution of “Not Effector” T cells (Treg/Tnull) in colorectal cancer. Front Immunol. (2017) 8:1900 10.3389/fimmu.2017.01900 29375559 PMC5770731

[34] Sugimoto MA Sousa LP Pinho V Perretti M Teixeira MM. Resolution of inflammation: what controls its onset? Front Immunol. (2016) 7:160. 10.3389/fimmu.2016.00160 27199985 PMC4845539

[35] Coffelt SB Wellenstein MD de Visser KE. Neutrophils in cancer: neutral no more. Nat Rev Cancer. (2016) 16:431–46. 10.1038/nrc.2016.52 27282249

[36] Coronella-Wood JA Hersh EM. Naturally occurring B-cell responses to breast cancer. Cancer Immunol Immunother. (2003) 52:715–38. 10.1007/s00262-003-0409-4 12920480 PMC11033039

[37] Mahmoud SM Lee AH Paish EC Macmillan RD Ellis IO Green AR. The prognostic significance of B lymphocytes in invasive carcinoma of the breast. Breast Cancer Res Treat. (2012) 132:545–53. 10.1007/s10549-011-1620-1 21671016

[38] Marsigliante S Biscozzo L Marra A Nicolardi G Leo G Lobreglio GB. Computerised counting of tumour infiltrating lymphocytes in 90 breast cancer specimens. Cancer Lett. (1999) 139:33–41. 10.1016/S0304-3835(98)00379-6 10408906

[39] Nzula S Going JJ Stott DI. Antigen-driven clonal proliferation, somatic hypermutation, and selection of B lymphocytes infiltrating human ductal breast carcinomas. Cancer Res. (2003) 63:3275–80. 12810659

[40] Olkhanud PB Damdinsuren B Bodogai M Gress RE Sen R Wejksza K. Tumor-evoked regulatory B cells promote breast cancer metastasis by converting resting CD4(+) T cells to T-regulatory cells. Cancer Res. (2011) 71:3505–15. 10.1158/0008-5472.CAN-10-4316 21444674 PMC3096701

[41] Zirakzadeh AA Marits P Sherif A Winqvist O. Multiplex B cell characterization in blood, lymph nodes, and tumors from patients with malignancies. J Immunol. (2013) 190:5847–55. 10.4049/jimmunol.1203279 23630345

[42] Yeong J Lim JCT Lee B Li H Chia N Ong CCH. High densities of tumor-associated plasma cells predict improved prognosis in triple negative breast cancer. Front Immunol. (2018) 9:1209. 10.3389/fimmu.2018.01209 29899747 PMC5988856

[43] Steinman RM Banchereau J. Taking dendritic cells into medicine. Nature. (2007) 449:419–26. 10.1038/nature06175 17898760

[44] Di Domizio J Demaria O Gilliet M. Plasmacytoid dendritic cells in melanoma: can we revert bad into good? J Invest Dermatol. (2014) 134:1797–800. 10.1038/jid.2014.155 24924760

[45] Simon S Labarriere N PD-1 expression on tumor-specific T cells: friend or foe for immunotherapy? Oncoimmunology. (2017) 7:e1364828 10.1080/2162402X.2017.1364828 29296515 PMC5739549

[46] Chaput N Louafi S Bardier A Charlotte F Vaillant JC Menegaux F. Identification of CD8+CD25+Foxp3+ suppressive T cells in colorectal cancer tissue. Gut. (2009) 58:520–9. 10.1136/gut.2008.158824 19022917

[47] Le Gallo M de la Motte Rouge T Poissonnier A Lavoue V Tas P Leveque J. Tumor analysis: freeze-thawing cycle of triple-negative breast cancer cells alters tumor CD24/CD44 profiles and the percentage of tumor-infiltrating immune cells. BMC Res Notes. (2018) 11:401. 10.1186/s13104-018-3504-5 29925435 PMC6011598

[48] Huang J Kerstann KW Ahmadzadeh M Li YF El-Gamil M Rosenberg SA. Modulation by IL-2 of CD70 and CD27 expression on CD8+ T cells: importance for the therapeutic effectiveness of cell transfer immunotherapy. J Immunol. (2006) 176:7726–35. 10.4049/jimmunol.176.12.7726 16751420 PMC1532931

[49] Luo Q Huang Z Ye J Deng Y Fang L Li X. PD-L1-expressing neutrophils as a novel indicator to assess disease activity and severity of systemic lupus erythematosus. Arthritis Res Ther. (2016) 18:47. 10.1186/s13075-016-0942-0 26867643 PMC4751645

[50] Kargl J Busch SE Yang GH Kim KH Hanke ML Metz HE. Neutrophils dominate the immune cell composition in non-small cell lung cancer. Nat Commun. (2017) 8:14381. 10.1038/ncomms14381 28146145 PMC5296654

[51] Shi C Pamer EG. Monocyte recruitment during infection and inflammation. Nat Rev Immunol. (2011) 11:762–74. 10.1038/nri3070 21984070 PMC3947780

[52] Yang J Zhang L Yu C Yang XF Wang H. Monocyte and macrophage differentiation: circulation inflammatory monocyte as biomarker for inflammatory diseases. Biomark Res. (2014) 2:1. 10.1186/2050-7771-2-1 24398220 PMC3892095

[53] Overgaard NH Jung JW Steptoe RJ Wells JW. CD4+/CD8+ double-positive T cells: more than just a developmental stage? J Leuko Biol. (2015) 97:31–8. 10.1189/jlb.1RU0814-382 25360000

[54] Ruhle PF Fietkau R Gaipl US Frey B. Development of a modular assay for detailed immunophenotyping of peripheral human whole blood samples by multicolor flow cytometry. Int J Mol Sci. (2016) 17:E1316. 10.3390/ijms17081316 27529227 PMC5000713

[55] Streitz M Miloud T Kapinsky M Reed MR Magari R Geissler EK. Standardization of whole blood immune phenotype monitoring for clinical trials: panels and methods from the ONE study. Transplant Res. (2013) 2:17. 10.1186/2047-1440-2-17 24160259 PMC3827923

[56] Verronese E Delgado A Valladeau-Guilemond J Garin G Guillemaut S Tredan O. Immune cell dysfunctions in breast cancer patients detected through whole blood multi-parametric flow cytometry assay. Oncoimmunology. (2016) 5:e1100791. 10.1080/2162402X.2015.1100791 27141361 PMC4839376

[57] Chen Z Hambardzumyan D. Immune microenvironment in glioblastoma subtypes. Front Immunol. (2018) 9:1004. 10.3389/fimmu.2018.01004 29867979 PMC5951930

[58] Quail DF Joyce JA. Molecular pathways: deciphering mechanisms of resistance to macrophage-targeted therapies. Clin Cancer Res. (2017) 23:876–84. 10.1158/1078-0432.CCR-16-0133 27895033 PMC5453188

[59] Ruffell B Coussens LM. Macrophages and therapeutic resistance in cancer. Cancer Cell. (2015) 27:462–72. 10.1016/j.ccell.2015.02.015 25858805 PMC4400235

[60] Elliott LA Doherty GA Sheahan K Ryan EJ. Human tumor-infiltrating myeloid cells: phenotypic and functional diversity. Front Immunol. (2017) 8:86. 10.3389/fimmu.2017.00086 28220123 PMC5292650

[61] Turrini R Pabois A Xenarios I Coukos G Delaloye JF Doucey MA. TIE-2 expressing monocytes in human cancers. Oncoimmunology. (2017) 6:e1303585. 10.1080/2162402X.2017.1303585 28507810 PMC5414874

[62] Brown MC Holl EK Boczkowski D Dobrikova E Mosaheb M Chandramohan V. Cancer immunotherapy with recombinant poliovirus induces IFN-dominant activation of dendritic cells and tumor antigen-specific CTLs. Sci Transl Med. (2017) 9:eaan4220. 10.1126/scitranslmed.aan4220 28931654 PMC6034685

