# Supplementary figures and images for: Examining Peripheral and Tumor Cellular Immunome in Patients With Cancer
Source: Front Immunol. 2019 Jul 31;10:1767. doi: 10.3389/fimmu.2019.01767 (PMC6685102; doi:10.3389/fimmu.2019.01767)

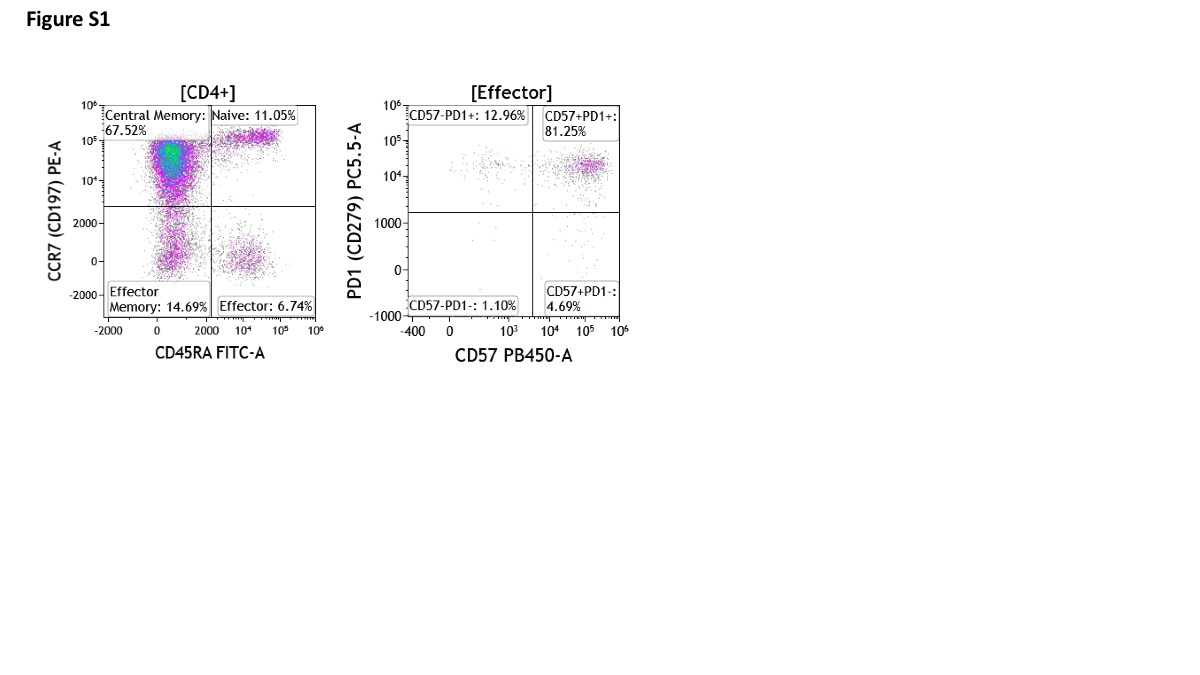

Supplement: Figure S1 — PD1 and CD57 expression on effector T cells in melanoma patients. CD4+ T cells that expressed CD45RA but lacked CCR7 were further examined for PD1 and CD57 cell surface expression. [file Image_1.JPEG]

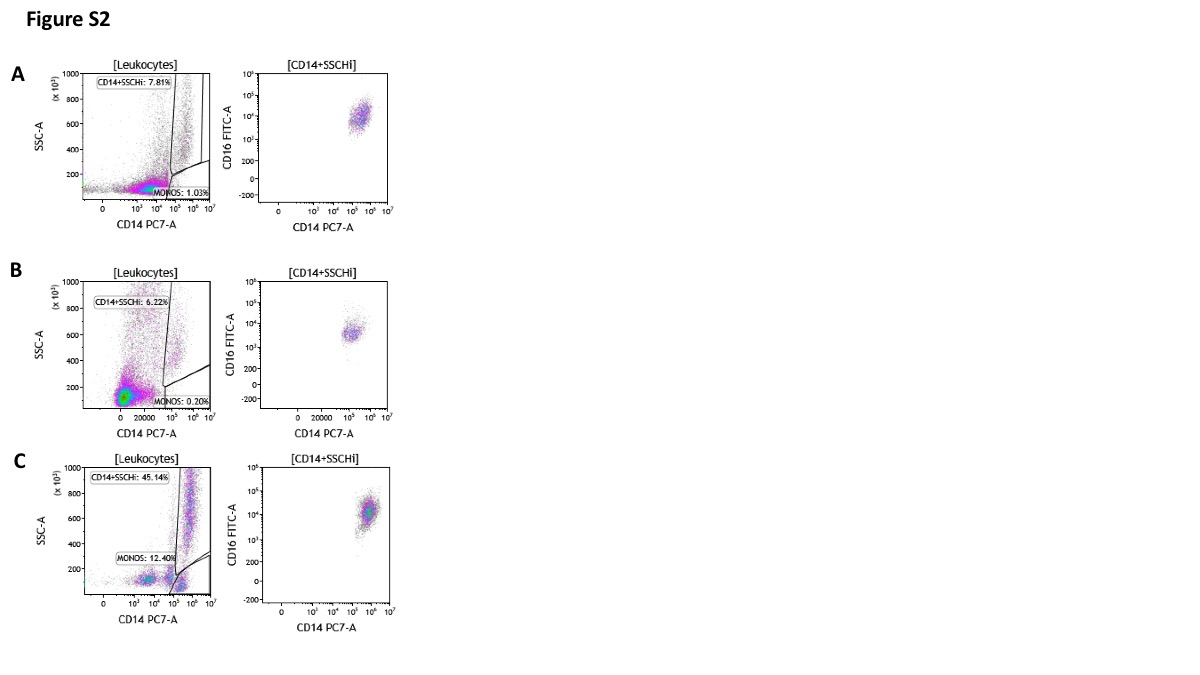

Supplement: Figure S2 — CD14+SSChi cells express CD14 and CD16. (A) melanoma; (B) breast cancer; (C) brain cancer. Tumors immune infiltrates from melanoma, breast, and brain tumor patients were analyzed as shown in Figures 8–10. All cells that were positive for CD14 with high SSC (side scatter) and distinct from blood monocytes were assessed for expression of CD14 and CD16. [file Image_2.JPEG]
