# Supplementary material for: Examining Peripheral and Tumor Cellular Immunome in Patients With Cancer
Source: Front Immunol. 2019 Jul 31;10:1767. doi: 10.3389/fimmu.2019.01767 (PMC6685102; doi:10.3389/fimmu.2019.01767)
Supplement: Table S1 — The frequency of leukocytes (as percentage of singlets), lymphocytes (as percentage of singlets), CD19+ B cells, CD3+ T cells, NK T cells and NK Cells (as percentage of all lymphocytes), CD4+ and CD8+ T cells (as percentage of CD3+ T cells), monocytes (as percentage of all leukocytes) and monocyte subsets (as percentage of all monocytes). Data shows average for all patients and low-high range. [file Data_Sheet_1.PDF]

Table S1:

**Basic Panel**	**Melanoma**		**Breast Cancer**		**Brain Cancer**	
**Cell Population**	Average	Range	Average	Range	Average	Range
Leukocytes	73.98	61.93-85.62	76.47	59.01-85.99	78.92	75-86.37
Lymphocytes	28.13	12.83-40.46	33.82	15.43-54.76	4.44	3.11-6.45
CD19+ B cells	6.74	1.3-11.58	6.79	1.88-14.97	21.74	15.81-28.40
CD3+ T cells	57.71	35.15-66.99	68.54	46.59-79.22	52.50	44.99-64.57
CD4+ T cells	76.31	67.85-82.69	65.28	37.03-77.11	72.12	65.96-77.41
CD8+ T cells	19.29	12.9-28.35	29.11	15.15-57.27	24.66	20.69-31.1
NK T Cells	12.98	6.9-16.69	9.26	2.08-14.87	6.64	4.06-12.26
NK Cells	17.75	5.74-33.82	12.98	4.41-28.24	16.20	9.35-25.67
Monocytes	11.06	3.25-16.18	8.50	6.51-11.69	3.50	1-5.92
CD14+CD16-	45.08	1.03-72.79	45.98	32.45-73.87	68.92	51.31-85.96
CD16+CD14-	9.61	4.05-14.15	7.78	1.59-12.81	1.11	0.36-2.39
CD16+CD14+	41.93	19.65-85.1	43.53	13.45-57.02	26.28	11.05-41.76

The frequency of leukocytes (as percentage of singlets), lymphocytes (as percentage of singlets), CD19+ B cells, CD3+ T cells, NK T cells and NK Cells (as percentage of all lymphocytes), CD4+ and CD8+ T cells (as percentage of CD3+ T cells), monocytes (as percentage of all leukocytes) and monocyte subsets (as percentage of all monocytes). Data shows average for all patients and low-high range.

Table S2:

T cell	Melanoma		Breast Cancer		Brain Cancer	
**Cell Population**	Average	Range	Average	Range	Average	Range
CD4+CD27+CD28-	0.22	0.0-0.45	0.66	0.01-2.86	0.14	0.0-0.56
CD4+CD27+CD28+	80.31	71.44-91.73	83.21	65.38-95.29	90.95	78.33-95.77
CD4+CD27-CD28-	5.21	0.1-10.53	8.69	0-27.07	2.68	0.0-10.69
CD4+CD27-CD28+	14.27	8.16-21.99	7.44	3.08-14.99	6.23	4.23-10.42
CD4+CD57+	9.76	2.65-17.68	10.01	1.7-21.86	6.29	1.57-19.17
CD4+Naive	22.15	11.05-32.09	26.09	10.94-39.77	25.91	9.03-51.48
CD4+Central Memory	52.68	40.5-67.52	51.95	35.88-62.96	61.62	43.59-69.34
CD4+Effector	5.07	0.56-7.61	2.58	0.3-5.35	1.20	0.33-3.33
CD4+Effector Memory	20.10	14.39-29.6	19.38	6.88-39.49	11.27	4.33-18.33
CD4+PD1+	22.88	0.04-45.91	36.88	23.62-48.12	26.87	19.69-38.19
CD8+CD27+CD28-	13.64	6.12-23.78	11.75	5.32-22.08	14.06	9.5-21.94
CD8+CD27+CD28+	46.76	37.15-55.13	56.95	25.76-88.49	57.89	41.84-81.79
CD8+CD27-CD28-	34.27	21.41-48.05	26.57	4.03-49.44	25.14	5.7-35.92
CD8+CD27-CD28+	5.33	2.16-8.41	4.74	1.24-15.26	2.91	0.46-5.28
CD8+CD57+	44.94	30.94-56.53	34.20	11.65-60.33	44.94	15.87-63.27
CD8+Naive	13.75	10.25-17.08	24.62	6.25-53.32	38.25	24.88-72.18
CD8+Central Memory	9.74	3.14-14.47	13.25	5.29-33.45	7.97	3.13-9.86
CD8+Effector	55.98	40-68.55	42.94	21.46-62.95	39.96	21.01-56.57
CD8+Effector Memory	20.53	12.14-39.78	19.19	9.4-36.16	13.82	3.69-29.59
CD8+PD1+	24.10	2.27-43	37.08	20.76-59.48	59.44	23.58-91.67

The frequency of CD4+ or CD8+ T cell subsets (as percentage of CD4+ or CD8+ T cells). Data shows average for all patients and low-high range.

Table S3:

T cell receptor	Melanoma		Breast Cancer		Brain Cancer	
**Cell Population**	Average	Range	Average	Range	Average	Range
HLA-DR+	38.41	30.75-51.09	41.09	28.15-63.47	23.44	15.54-30.85
TCRαβ+	97.95	95.29-99.23	93.88	82.91-98	94.95	93.18-98.46
TCRγδ+	1.69	0.69-3.32	6.01	1.92-16.98	4.49	1.28-6.82
TCRγδ+TCRαβ+	0.13	0.02-0.35	0.07	0.01-0.15	0.47	0.0-1.84
TCRαβ-TCR γδ-	0.24	0.03-1.04	0.05	0.01-0.09	0.09	0.0-0.24
CD4 TCRαβ+	73.22	61.79-88.83	67.83	38.13-82.03	75.15	71.22-78.14
CD8 TCRαβ+	22.45	7.37-33.54	28.02	14.74-58.96	23.17	19.25-27.49
Vδ1+	32.62	17.4-45.39	40.38	6.11-90.91	14.15	6.67-24.24
Vδ1+Vδ2+	1.31	0.0-5.92	0.17	0-0.49	0.00	0.00
Vδ1-Vδ2-	12.62	6.78-28.57	12.12	3.16-36.31	3.81	1.67-7.69
Vδ2+	53.44	34.29-75.81	47.33	5.93-77.7	82.05	72.73-91.67

The frequency of HLA-DR+ T cells (as percentage of CD3+ T cells), TCRαβ and TCRγδ T cells (as percentage of CD3+ T cells), CD4+TCRαβ+ and CD8+TCRαβ+ T cells (as percentage of TCRαβ+ T cells), Vδ1-Vδ2-, Vδ1+, Vδ2+ and Vδ1+Vδ2+ (as percentage of all TCRγδ+ T cells). Data shows average for all patients and low-high range.

Table S4:

**Regulatory T cell**	**Melanoma**		**Breast Cancer**		**Brain Cancer**	
**Cell Population**	Average	Range	Average	Range	Average	Range
CD3+CD4+	46.17	40.39-51.94	44.31	38.59-49.68	35.37	33.24-37.97
CD4+CD39+	10.92	5.92-15.92	29.83	0.98-98.66	12.61	8.25-15.65
CD4+CD25+	7.13	6.27-7.98	10.35	5.83-18.90	13.87	13.63-14.06
FoxP3+Helios+	1.83	0.83-2.83	5.91	3.24-9.00	4.78	0.26-8.31

The frequency of CD3+CD4+ T cells (as percentage of all lymphocytes), CD39+, CD25+ or FoxP3+Helios+ subsets (as percentage of CD4+ T cells). Data shows average for all patients and low-high range.

Table S5:

Granulocytes	Melanoma		Breast Cancer		Brain Cancer	
**Cell Population**	Average	Range	Average	Range	Average	Range
CD294+	2.50	0.56-4.81	1.19	0.31-2.94	0.02	0-0.04
Basophils	31.98	17.7-44.79	43.54	7.05-87.41	*	*
Eosinophils	60.71	44.27-80.67	49.54	6.29-87.99	*	*
CD15+	40.88	28.68-58.94	50.86	30.07-73.13	87.92	82.95-92.32
CD62L-	10.67	4.74-19.39	17.04	4.53-78.51	6.59	2.23-12.54
PDL1+	23.03	11.77-49.65	21.00	5.04-31.24	1.04	0.12-2.22

The frequency of CD294+ cells (as percentage of singlets), basophils and eosinophils (as percentage of all CD294+ cells), CD15+ cells (as percentage of all cells without CD294+ cells), CD62L- (as percentage of CD15+ cells), PDL1+ (as percentage of CD15+ cells). Data shows average for all patients and low-high range.

Table S6:

**B cells**	**Melanoma**		**Breast Cancer**		**Brain Cancer**	
**Cell Population**	Average	Range	Average	Range	Average	Range
CD19+ B cells						
Naïve B cells	46.22	21.43-63.57	59.95	11.46-80.90	46.63	40.99-54.47
Marginal Zone B cells	19.49	13.71-26.26	7.16	2.03-19.43	26.06	19.07-37.04
IgD-CD27-	6.72	2.43-12.01	7.68	3.47-12.48	6.09	3.11-11.63
IgD-CD27+	27.57	8.72-48.38	25.21	9.25-63.89	21.22	9.57-31.12
CD21^{low}CD38^{low}	10.99	3.03-19.52	6.39	1.26-30.9	9.83	2.88-26.67
IgM-IgD-	31.31	7.39-58.44	31.04	14.63-71.88	24.66	11.96-31.68
Class-Switched Memory B cells	44.58	30.30-54.44	51.05	27.45-70.65	43.22	26.98-60.08
CD27+CD38+	36.75	23.74-52.66	25.88	11.78-56.86	34.97	20.65-60.32
CD27-CD38-	10.04	3.77-13.56	12.31	0-22.87	11.69	3.17-27.44
CD27-CD38+	8.64	6.17-14.39	10.76	2.55-27.52	10.12	5.53-16.13
Plasmablasts	8.82	0.78-27.5	13.72	2.24-47.06	6.11	1.94-15.87
IgM+IgD+	62.91	34.74-90.16	66.56	21.18-83.28	73.30	67.25-86.03
Class-Unswitched Memory B cells	35.18	20.83-57.01	15.94	1.78-57.38	40.38	32.97-46.82
IgM+CD27+CD38high	1.15	0.0-2.95	4.21	0.00-16.67	0.87	0.19-1.67
IgM+CD27-CD38dim	59.38	39.25-78.63	58.37	4.55-90.84	53.44	45.72-60.54
IgM+CD27-CD38high	4.29	0.29-15.58	21.48	0.00-76.14	5.32	1.21-9.81
CD38+	63.05	41.12-78.93	79.60	42.62-93.23	57.72	51.57-65.95
Transitional B cells	4.84	0.12-14.44	23.86	0.00-93.43	8.18	1.84-20.24

The frequency of B cell subsets (as percentage of CD19+ B cells), class-switched memory B cells, CD27-CD38-, CD27+CD38+, CD38+CD27- (as percentage of all IgM-IgD- CD19+ B cells), class-unswitched memory B cells, IgM+CD27+CD38^{high}, IgM+CD27-CD38^{dim}, IgM+CD27-CD38^{dim} (as percentage of all IgM+IgD+ CD19+ B cells), transitional B cells (as percentage of all CD38+ CD19+ B cells). Data shows average for all patients and low-high range.

Table S7:

Dendritic cells	Melanoma		Breast Cancer	
**Cell Population**	Average	Range	Average	Range
Lin-HLADR+	0.65	0.14-1.36	1.30	0.20-1.92
pDCs	13.99	5.81-29.62	4.05	1.22-32.20
mDCs	27.75	4.15-47.01	65.85	12.10-74.45
CD16+ DCs	71.62	45.6-90	87.58	53.11-94.37
CD11c+Clec9A+ CD16-	4.09	0.76-10	0.24	0.00-3.79
CD11c+CD1c+CD16-	23.58	8.14-47.25	6.39	1.48-29.84

The frequency of Lin-HLADR+ cells (as percentage of singlets), pDCs (as percentage of Lin-HLADR+ cells), mDCs (as percentage of Lin-HLADR+ cells), CD16+, CD11c+Clec9A+ CD16- and CD11c+CD1c+CD16- subsets (as percentage of mDCs). Data shows average for all patients and low-high range.

Table S8:

Basic Panel	Melanoma		Breast Cancer		Brain Cancer	
**Cell Population**	Average	Range	Average	Range	Average	Range
Leukocytes	17.31	2.02-39.12	28.53	1.59-75.21	2.36	0.44-4.61
Lymphocytes	12.22	1.3-23.9	14.69	0.17-48.47	0.73	0.12-1.6
CD19+ B cells	17.26	3.16-36.38	23.05	2.17-63.19	31.76	14.2-72.2
CD3+ T cells	76.44	54.77-89.95	56.07	17.93-78.96	18.22	9.71-26.54
CD4+ T cells	44.58	15.49-63.16	59.15	31.41-77.94	48.44	34.7-59.16
CD8+ T cells	49.04	33.29-76	36.47	20.2-61.78	41.14	30.28-52.96
NK T Cells	2.12	0.3-4.17	7.95	2.44-21.95	12.02	8.74-18.85
NK Cells	3.14	0.3-7.35	5.84	0-10.44	28.49	14.23-50.45
Monocytes	3.53	0.98-6.55	4.92	0.06-26.4	6.29	0.38-12.55
CD14+CD16-	25.37	5.41-60.78	24.29	0-58.33	57.88	51.43-71.7
CD16+CD14-	0.00	0.00	0.12	0-0.77	0.00	0.00
CD16+CD14+	73.45	39.22-91.89	69.34	26.67-100	40.47	27.02-48.57

The frequency of leukocytes (as percentage of singlets), lymphocytes (as percentage of singlets), CD19+ B cells, CD3+ T cells, NK T cells and NK Cells (as percentage of all lymphocytes), CD4+ and CD8+ T cells (as percentage of CD3+ T cells), monocytes (as percentage of all leukocytes) and monocyte subsets (as percentage of all monocytes). Data shows average for all patients and low-high range.

Table S9:

T cell	Melanoma		Breast Cancer		Brain Cancer	
**Cell Population**	Average	Range	Average	Range	Average	Range
CD4+CD27+CD28-	1.00	0-2.58	0.53	0-2.08	1.21	0.41-2.28
CD4+CD27+CD28+	76.24	49.72-89.27	50.90	11.61-79.75	67.18	47.53-86.11
CD4+CD27-CD28-	2.14	0.09-5.79	1.48	0-4.17	2.93	1.23-5.7
CD4+CD27-CD28+	20.63	8.89-41.91	47.09	18.35-87.1	28.68	11.11-44.49
CD4+CD57+	12.82	0.64-51.67	7.87	0-44.44	14.52	6.84-29.66
CD4+Naive	0.47	0-1.22	5.14	0-22.22	3.51	0.76-7
CD4+Central Memory	67.68	40.1-91.44	80.38	62.71-94.01	49.60	19.39-79.42
CD4+Effector	0.03	0-0.14	0.00	0-0	0.00	0.00
CD4+Effector Memory	31.82	8.39-59.81	14.47	5.56-37.29	46.88	13.58-79.85
CD4+PD1+	52.30	25.89-64.99	76.91	60.22-93.75	91.56	87.04-95.06
CD8+CD27+CD28-	11.17	5.28-20.96	10.84	2.62-28.34	11.94	5.43-16.9
CD8+CD27+CD28+	66.34	52.6-77.01	48.74	22.06-71.16	55.26	46.09-70.42
CD8+CD27-CD28-	10.48	4.74-18.6	15.89	5.06-43.09	11.94	5.63-17.52
CD8+CD27-CD28+	12.01	9.17-18.9	24.53	6.51-50	20.85	7.04-32.61
CD8+CD57+	31.95	17.51-54.93	24.24	5.73-62.44	17.32	5.66-31.54
CD8+Naive	0.41	0-1.16	6.56	0.97-27.45	12.44	0.54-18.48
CD8+Central Memory	56.78	28.38-76.76	59.20	7.9-81.53	41.28	25.34-71.74
CD8+Effector	0.43	0-1.2	8.03	0-63.69	7.90	0.36-22.54
CD8+Effector Memory	42.37	23.03-70.74	26.22	17.5-50	38.38	9.42-73.32
CD8+PD1+	48.02	38.21-55.02	67.32	28.32-84.76	89.12	77.46-96.38

The frequency of CD4+ or CD8+ T cell subsets (as percentage of CD4+ or CD8+ T cells). Data shows average for all patients and low-high range.

Table S10:

BASIC	Pre-Chemo		Post-Chemo		Post-Surgery	
**Cell Population**	Average	Range	Average	Range	Average	Range
Leukocytes	77.37	63.84-88.26	81.75	69.52-92.18	78.64	69.85-89.31
Lymphocytes	37.03	20.55-64.49	54.23	41.5-69.29	49.33	41.99-55.16
CD19+ B cells	18.58	6.3-27.41	8.89	7.65-9.71	13.19	4.87-21.18
CD3+ T cells	67.91	57.06-78.64	72.30	61.29-81.09	73.25	64.92-80.35
CD4+ T cells	71.58	65.59-81.95	70.87	59.49-84.73	77.33	72.99-83.7
CD8+ T cells	25.17	16.02-33.07	25.32	13.02-32.6	19.85	14.1-23.5
NK T Cells	5.37	2.24-7.27	7.57	2.07-15.38	4.94	1.61-6.78
NK Cells	7.61	6.93-7.98	9.91	6.07-13.49	6.44	5.04-7.21
Monocytes	7.31	5.02-8.56	6.62	4.85-8.1	7.58	6.06-10.58
CD14+CD16-	42.73	36.88-49.6	41.09	35.42-50.14	47.97	41.97-53.04
CD16+CD14-	4.62	2.35-8.33	10.32	6.79-14.72	6.60	3.93-9.88
CD16+CD14+	47.11	38.33-57.26	43.83	37.59-48.36	41.59	38.64-45.95

The frequency of leukocytes (as percentage of singlets), lymphocytes (as percentage of singlets), CD19+ B cells, CD3+ T cells, NK T cells and NK Cells (as percentage of all lymphocytes), CD4+ and CD8+ T cells (as percentage of CD3+ T cells), monocytes (as percentage of all leukocytes) and monocyte subsets (as percentage of all monocytes). Data shows average for all patients and low-high range. The patients presented in this study had varying degrees of response to chemotherapy. Patient 1 exhibited a complete pathologic response, patient 2 had a partial response to chemotherapy with sub-millimeter foci of residual tumor in the tumor bed and patient 3 had a minimal response to chemotherapy.
